# Reliability of case definitions for public health surveillance assessed by Round-Robin test methodology

**DOI:** 10.1186/1471-2458-6-129

**Published:** 2006-05-10

**Authors:** Gérard Krause, Bonita Brodhun, Doris Altmann, Hermann Claus, Justus Benzler

**Affiliations:** 1 Department of Infectious Disease Epidemiology, Robert Koch Institute, Seestr. 10, 13353 Berlin, Germany

## Abstract

**Background:**

Case definitions have been recognized to be important elements of public health surveillance systems. They are to assure comparability and consistency of surveillance data and have crucial impact on the sensitivity and the positive predictive value of a surveillance system. The reliability of case definitions has rarely been investigated systematically.

**Methods:**

We conducted a Round-Robin test by asking all 425 local health departments (LHD) and the 16 state health departments (SHD) in Germany to classify a selection of 68 case examples using case definitions. By multivariate analysis we investigated factors linked to classification agreement with a gold standard, which was defined by an expert panel.

**Results:**

A total of 7870 classifications were done by 396 LHD (93%) and all SHD. Reporting sensitivity was 90.0%, positive predictive value 76.6%. Polio case examples had the lowest reporting precision, salmonellosis case examples the highest (OR = 0.008; CI: 0.005–0.013). Case definitions with a check-list format of clinical criteria resulted in higher reporting precision than case definitions with a narrative description (OR = 3.08; CI: 2.47–3.83). Reporting precision was higher among SHD compared to LHD (OR = 1.52; CI: 1.14–2.02).

**Conclusion:**

Our findings led to a systematic revision of the German case definitions and build the basis for general recommendations for the creation of case definitions. These include, among others, that testable yes/no criteria in a check-list format is likely to improve reliability, and that software used for data transmission should be designed in strict accordance with the case definitions. The findings of this study are largely applicable to case definitions in many other countries or international networks as they share the same structural and editorial characteristics of the case definitions evaluated in this study before their revision.

## Background

Case definitions have been recognized to be important elements of public health surveillance systems [1]. They are to assure comparability and consistency of surveillance data and have crucial impact on the sensitivity and the positive predictive value of a surveillance system. The World Health Organization has been encouraging the use of case definitions to make surveillance data comparable between countries. One of the first case definitions used for national disease reporting was the case definition for AIDS published by the Centers for Disease Control and Prevention (CDC) in 1982 [2]. In 1985 Sacks published a survey among all 50 US states, Puerto Rico, and Washington, DC, that revealed important variations in the case definitions between the different states, and concluded the necessity to unify case definitions if surveillance data between states are to be compared [3]. In 1990 the CDC in collaboration with the Council of State and Territorial Epidemiologists published an edition of case definitions for public health surveillance [4,5].

Since then case definitions have become an important tool of other national surveillance systems and international surveillance networks. Koo and colleagues have analyzed surveillance data for Cholera in Latin America and have described the importance of uniform case definitions to make data comparable between countries [6]. In 2003 the European Union (EU) case definitions for the European networks have reached obligatory status for the member states reporting to the EU [7]. During the SARS epidemic the case definition had a major impact on whether and how countries were considered affected or not, resulting in severe political and economic consequences for a number of countries [8].

Coggon and colleagues have demonstrated the difficulties of determining optimal case definitions if a satisfactory diagnostic gold standard is lacking [9]. In sharp contrast to the importance of case definitions hardly any research has been published on the performance of surveillance case definitions. Studies are rare on how local health departments and other health professionals are able to understand case definitions and to what extent case definitions are unambiguous enough to really assure reliability. To our knowledge, the only publication investigating this issue was focused on case definitions for nosocomial infections: Gastmeier and colleagues had investigated how uniform the case definitions of the nosocomial infections surveillance system in Germany had been applied by different investigators using a set of 60 case studies [10]. Due to the general importance of case definition for public health surveillance and the current need for harmonization in international surveillance systems we conducted a systematic evaluation of the national case definitions with the objective to identify general as well as specific criteria and recommendations for improvement of case definitions.

## Methods

### Notification and reporting procedure

Germany is a federal republic with 16 states subdivided into 440 counties. As in many countries the local (county) health departments (total number: 425) are the primary recipients of infectious disease notifications made by physicians and laboratories. Local health departments verify the incoming notifications and assess the need for public health action. Local health departments use one of five software products on the market to classify the case reports according to the national edition of case definitions and to report these cases electronically to the state health department. From there the report is being forwarded to the Robert Koch Institute (RKI), the federal institution in charge of national infectious disease surveillance in Germany [11].

### Introduction of case definitions

The edition of national case definitions for all notifiable infectious diseases was introduced in Germany in 2001, following the implementation of a new law to control infectious diseases (Infektionsschutzgesetz, IfSG) [12-14]. The IfSG determines the set of diseases and pathogens to be notified by physicians and laboratories throughout the Federal Republic of Germany. The five eastern states, which formerly belonged to the Democratic Republic of Germany (East Germany) and the State of Berlin have enacted complementary rules that make certain diseases additionally notifiable within the state jurisdiction, that are not notifiable in all of Germany.

The case definitions were developed by the RKI, using the delphi method including the expertise of state epidemiologists, national reference laboratories and medical and scientific associations for the specific diseases. The case definitions for infectious conditions under public health surveillance published by the CDC were also taken into account [5,15]. After having published the IfSG case definitions in the fall of 2000 to be implemented with the beginning of 2001 the RKI also published additional case definitions in January 2002 for some of the diseases exclusively notifiable in the eastern states jurisdictions [11,16]. From June 2002 to September 2003 we had conducted a systematic evaluation of the case definitions with the purpose to revise them by the end of 2003.

### Structure and classification of case definitions

The German case definitions are divided into three types of evidence: Clinical picture, laboratory detection, and epidemiological confirmation. The types of evidence are specifically defined for each disease (see table 1). Based on whether or not requirements for these three types of evidence are fulfilled a case is classified into five categories. In the revised 2004 edition of case definitions these categories are named: A) clinically diagnosed illness (neither epidemiologically nor laboratory-confirmed), B) clinically and epidemiologically confirmed illness (not laboratory-confirmed), C) clinically and laboratory-confirmed illness, D) laboratory-detected infection not fulfilling clinical criteria, E) laboratory-detected infection with unknown clinical picture. (In the 2001 edition of case definitions these five categories were named slightly differently)

For most notifiable diseases only categories B, C, D and E are reportable from the local health department to the next level, requiring at least laboratory detection of the pathogen or epidemiological confirmation. For some exceptions (e.g. tuberculosis, polio, measles, Creutzfeldt-Jakob disease), cases are also reported from the local health department to the next level if category A – the clinical picture alone – is fulfilled.

### Round-Robin test

In June 2002 we conducted a Round-Robin test in analogy to the established quality control procedure of laboratories and other testing units [17]. Round-Robin tests are mainly used in proficiency tests in order to determine laboratory performance by means of comparing tests on identical items by two or more laboratories in accordance with predetermined conditions [18].

We asked each local and state health department to classify a selection of 68 written case examples on the basis of the case definitions that were implemented in 2001 (2002 respectively for disease only notifiable in East German States). While proficiency tests generally intent to assess the ability of laboratories in finding identical results, we applied this method to assess to which extend the case definitions were unambiguous enough to assure identical classification by the health departments.

#### Definitions of outcome variables

We applied four different outcome variables in our analysis:

1) Disease identification: A disease was defined as being correctly identified if the participant of the Round-Robin test was able to identify the correct disease of the case example.

2) Case categorization: A case example was considered correctly categorized if the participant classified the case example with the correct disease and the correct case definition category as defined in the gold standard.

3) Reporting: The decision on reportability was considered correct if a case that should have been reported to the next level would have been forwarded according to the case definition category, given that the correct disease was identified. Inversely decision on reporting was also seen to be correct if a case that should not have been reported to the next level was in fact classified in a way that the case would have been held back. However, cases forwarded with wrong disease identification (see above) were a priori considered incorrect. Thus reporting was based on the question whether the case needed to be forwarded to the state level or not, which is a direct result of the disease identification and the case definition category. Sensitivity of reporting was defined as the number of cases that would have been correctly forwarded divided by the number of cases that should have been forwarded according to the gold standard. The positive predictive value of reporting was defined as the number of cases that should have been forwarded among those that would have been forwarded. Precision of reporting is defined as the number of cases that would have been either correctly forwarded to the state level, or would have been correctly held back at the local health department level, divided by the total number of case examples. Unless stated otherwise, reporting precision was the outcome parameter used in the following analysis.

4) Clinical classification: To specifically assess the effect of different styles in formulating case definitions, a fourth outcome variable was used. The clinical classification was considered correct if the part regarding the clinical picture was classified according to the gold standard, regardless whether other parts of the case definition were correctly classified or not. This analysis was done to compare case definitions with narrative description of the clinical picture (as in all former IfSG case definitions) to case definitions with a more explicit check-list format of clinical criteria, that was implemented for diseases additionally notifiable in specific states and for the new IfSG case definitions.

#### Case examples

The case examples consisted in facsimile excerpts of one or more of the following sources: laboratory report form, physician form, and protocol of the patient interview [see additional file 1]. The case examples were created based on real cases that have come to the attention of the RKI in the quality control process and in the information service hotline that the RKI is offering to the health departments. The case examples were pre-tested among epidemiologists within the RKI and among epidemiologists and public health nurses in the state and local health departments.

#### Selection and distribution of case examples

A total of 68 case examples for 17 different diseases were created. In order to limit the time needed to classify the case examples, each local health department in West Germany (including Berlin) received four different case examples each for four different notifiable diseases resulting in 16 case examples to be classified. Local health departments in East Germany (excluding Berlin) received an additional set of four case examples for one of four diseases additionally notifiable in East German states, resulting in a total of 20 case examples.

In order to stratify the case examples of the 17 diseases among the local health departments, we created eight sets of case examples, as shown in table 2. Sets A to D were randomly distributed among local health departments in each West German state; sets E to H were randomly distributed among local health departments of each East German state. As shown in table 3, the *Salmonella *case examples appeared in all eight sets, in order to have the possibility to compare between local health departments and other determinants based on identical case examples.

Epidemiologists in state health departments participating in the study received set A (West Germany) or set E (East Germany). Epidemiologists at the RKI, not involved in designing the case examples, were asked to fill out all 68 case examples. For the analysis the variable "group" was defined as indicated in table 2 in order to control for a possible allocation bias of participants.

#### Gold standard definition

After the data of the respondents had been analyzed, the classification originally intended while creating the case definition, was challenged with the results of the respondents. Three epidemiologists then reassessed each individual case example and re-examined whether the classification originally intended was still justified. Based on this process the gold standard was defined for each case example.

#### Quantitative analysis

We compared the responses to the established gold standard and stratified by the following variables: health department being in an East German versus a West German state, disease of the case example, whether or not physicians participate in routine quality control of case reports (versus this being done exclusively by public health nurses), institutional level (local health department, state health department, RKI), acceptance and style of case definitions (check-list vs. text) and software used at local health department. Because of the selection and distribution of case examples described above, we conducted the individual analyses for each group. After univariate analysis we conducted a multivariate analysis using SPSS 13.0 for Windows (Version 13.0.1).

### Qualitative analysis

The distribution of the classifications was compared to the gold standard, in order to identify common discrepancies. Based on these discrepancies we identified which part of the case definition was affected and identified specific aspects of the case definitions that had repeatedly been interpreted differently by the participants, indicating failure of the case definition to be unambiguous and reliable. These aspects were then summarized in order to deduct commonalities which could then lead to specific recommendations on how to improve this particular case definition and also on how to improve formulation of case definitions in general.

### Survey

In May 2002 – simultaneously with the Round-Robin test – we conducted a written survey addressed to all 425 local health departments in Germany. Among various questions on the structure and equipment of the local health departments, and their experiences with the new IfSG, we also asked about the profession of the person who had actually filled the questionnaire and about his or her attitudes and experiences towards the case definitions.

## Results

### Study population

We received completed forms from 396 (93%) of 425 local health departments. Additionally, 30 epidemiologists from all 16 states and 18 epidemiologists within the RKI had completed the forms. This resulted in a total of 7870 classifications of case examples.

The survey was completed by 400 (94%) of the 425 health departments.

### Quantitative analysis of Round-Robin test

The overall result of the Round-Robin test shows that in 7003 case examples (89.0% of all 7870 classified case examples) the correct disease was identified (disease identification). In 4073 case examples (51.8%) the participants classified the case in the expected case definition category (case categorization). In 5042 case examples (64.1%) the correct disease was classified such that the case would have been reported and in 4847 case examples (61.6%) the clinical categorization was correct. Of 4291 case examples that were reportable, 3860 (90.0%) were classified such that they would have been reported (reporting sensitivity); of 5042 case examples that would have been reported to the state health department, 3860 (76.6%) were actually reportable (positive predictive value of reporting), while 1182 (23.4%) should have been excluded from reporting. Of all 7870 case examples the precision of reporting was 79.5%.

#### Group-wise multivariate analysis

The multivariate analysis was limited to data from the local health departments and without additional case examples for the East German states (n = 5995). Only statistically significant associations are mentioned in the following.

#### Disease

The disease of the case examples was for all groups significantly associated with reporting (p < 0.001 in group 1, 2 and 4, p = 0.022 in group 3).

#### Software used at local health department

Local health departments using the RKI-software showed a higher chance to identify the disease (disease identification) of the case example according to the gold standard compared to health departments using any of the commercially available software programs (group 2: OR = 1.85, CI: 1.20 – 2.84 and group 3: OR = 1.76, CI: 1.17 – 2.66). Additionally health departments in group 3 using the RKI software had a higher chance of classifying case examples (clinical classification) according to the gold standard (OR = 1.32, CI: 1.01 – 1.70).

#### East Germany versus West Germany

Analysis of case examples used in both East and West Germany showed that local health departments in East Germany had a lower chance of identifying the disease correctly compared to West German local health departments in group 1 (OR = 0.40, CI: 0.27 – 0.58), of identifying the case definition category correctly in group 2 (OR = 0.76, CI: 0.58 – 1.00) and of correctly reporting in group1 (OR = 0.73, CI: 0.54 – 0.99).

#### Participating professions

Local health departments where the physician was involved in applying the case definition in the daily routine, showed a lower chance of agreement in disease identification in group 1 (OR = 0.61, CI: 0.41 – 0.89) and a higher chance of agreement in reporting in group 4 (OR = 1.36, CI: 1.02 – 1.81).

#### Attitudes towards case definitions

Local health departments that stated that case definitions were a valuable tool had higher rates of agreement with the gold standard for disease identification (OR = 2.09, CI: 1.16 – 3.75) and case categorization (OR = 1.71, CI: 1.10 – 2.65) in group 1.

#### Summarized multivariate analysis

In order to assess whether there might have been a bias in allocating participating health departments to specific groups of case examples we made a separate analysis exclusively with the 4 *Salmonella*-like case examples which all groups had in common. The analysis among the 1508 classified case examples showed no significant association between group and reporting, suggesting no evidence for allocation bias.

In the summarized model, in which the responses of all groups were analyzed, the disease of the case examples was significantly associated with reporting (p < 0.001). Local health departments where a physician was involved in applying the case definition in the daily routine, showed a lower chance to identify the disease correctly (OR = 0.82, CI: 0.68 – 0.99). Local health departments in East Germany had a lower chance to identify the disease correctly (OR = 0.74, CI: 0.62 – 0.88) and of reporting correctly to the next level compared to West German local health departments (OR = 0.84, CI: 0.73 – 0.96).

#### Comparing the diseases

For the outcome variable 'reporting' the case examples of *Salmonella *had higher rates of agreement with the gold standard compared to case examples of all other diseases. For this reason separate analyses were done comparing the different diseases by using *Salmonella *cases examples as the reference variable adjusted for East/West. Based on the magnitude of the odds ratios, we found that the examples for CJD (OR = 0.012, CI: 0.008 – 0.017) and Polio (OR = 0.008, CI: 0.005 – 0.013) had the lowest chance of reporting precision compared to *Salmonella *case examples. Details are shown in table 1.

#### Narrative format versus check-list format

The classification of the clinical picture, one element of the case definition, was separately analyzed in a model including exclusively data from East German health departments (n = 2019) and RKI (n = 1016). These were the only participants exposed to case examples of disease nationally reportable (with narrative description of the clinical picture) and to case examples of diseases only notifiable in East German states (check-list format). The results of the univariate analysis show, that agreement in case classification with the gold standard was more than three times as high when the respective case definitions had listed the clinical criteria in a check-list format instead of a narrative description (OR = 3.08, CI: 2.47 – 3.83).

#### Administrative level

The administrative level at which the respondents worked, was significantly associated with the outcome reporting. For the analysis we used all cases of set A and set E (without the additionally diseases for the East German States, n = 2213). Adjusted for the diseases the chance of correct reporting to the next level was 1.5 times higher in cases done by state level staff compared to those done by local health department staff (OR = 1.52, CI: 1.14 – 2.02).

### Qualitative results

The following observations have been made in the qualitative analysis of the responses:

• The concept of epidemiological confirmation was not well understood. For example travel in endemic countries was equivocally seen as an epidemiological confirmation (e.g. haemorrhagic fever and travel to Egypt). Re-evaluation of the case definitions showed that in fact there was only a vague definition of the epidemiological confirmation.

• Participants appeared to have difficulties in deciding whether all clinical signs and symptoms mentioned in the case definition had to be existent in a case, or whether they were only listed as descriptive examples.

• Case examples of diarrheal disease without any evidence of a specific pathogen, were frequently classified as salmonellosis.

• Laboratory findings with only one elevated antibody value in serum were repeatedly classified as laboratory detection although the case definition required a rise in antibody level.

• In some case definitions detection of the pathogen is only accepted if the detection was done in specific materials (normally sterile material such as blood for detecting *N. meningitidis*). This limitation was frequently neglected.

• Some of the information in the case definition intended to serve as additional background information was mistakenly used as selection criteria (e.g. statement that clinician described rash as "very typical" for measles, but fever was missing).

### Survey results

When asked about the availability of the case definitions, 395 (99%) of 398 local health departments responded that the case definition were accessible at the work place. The case definitions were seen as useful by 377 (95%) of 397 health departments who answered this question and not useful by 20 (5%). The clarity of the individual sections of the case definitions was rated differently: The section on the clinical picture of the case definitions was seen as unambiguous in all case definitions by 72 respondents (18%), in the majority of case definitions by 305 (76%), in the minority by 20 (5%), and in none of the case definitions by one (0.3%) of the respondents (n = 398 respondents). The section on the laboratory confirmation of the case definitions was seen as unambiguous in all case definitions by 137 respondents (34%), in the majority of case definitions by 248 (62%), and in the minority by 11 (3%) (n = 396).

Three-hundred and three (87%) of 347 health departments stated that case classifications were done exclusively or primarily by public health nurses. With respect to the case examples presented to the participants, 220 (55%) of 396 respondents (from the local health departments) stated that the case examples were realistic.

## Discussion

The results of our evaluation have shown that although case definitions may appear to be clearly defined, they may be interpreted quite differently by their users, which may result in severe misclassifications and reduced sensitivity and positive predictive value. This study is believed to be the first to systematically assess these effects quantitatively on a large scale, covering 396 (93%) of 425 local health departments in Germany providing at the same time clear evidence on how case definitions can be improved.

The sensitivity and the positive predictive value calculated from this Round-Robin test does not have the intention to represent the respective values of the real surveillance data, these values serve as comparative measurements within the study. It must be kept in mind that we created the case examples specifically to identify need for improvement of the case definitions. Therefore the majority of case examples were intentionally characterized by borderline constellations, meant to represent realistic challenges to the case definition and its user. We also intentionally included rare diseases with high public health importance. The fact that 56% of the participants perceived the case examples to be realistic, indicates that daily routine might generally confront health departments with case reports that might be easier to classify. This explains why case examples of polio (no reported cases since 1998) and CJD (approx. 80 cases per year) had extremely lower rates of agreement compared to *Salmonella *case examples (approx. 57.000 cases per year) [19,20].

Also the complexity of the case definition itself is likely to affect reporting precision. Unfortunately much of the complexity of the case definition is a result of methodological limitations of available laboratory tests and cannot be influenced. The case definition system with its three different types of evidence leading to five different categories may appear very complex and less intuitive that the classical categories of "suspect", "probable" and "confirmed". The detailed differentiation of the German case definitions however enables us to apply computer algorithms in order to translate these to the EU case definitions and thus make the data compatible to the standards of various European surveillance networks and to WHO reports.

Reassessment of the gold standard after receipt of the responses resulted in modifications of 5 of the 68 case examples. This procedure took place in an initial review process of gold standard before the actual analysis was done. We believe it was legitimate and necessary in order to correct for biases caused by unforeseen ambiguity of the case examples.

The software used at the local health department was significantly associated with the quality of the data in only some subgroups and outcomes. Apparently the software is not a very strong determinant in the given study design, although our experience in implementing the electronic surveillance system in Germany showed that commercially available software products often do not fully implement the standards published by the RKI for data transmission software or they do so with a delay of several years [21].

The other interesting finding is that the administrative level of the participants was significantly associated with the outcome: Participants from state health departments had a significantly higher rate of agreement with the reporting gold standard than the participants from local health departments. This might be explained by the fact that staff at the state level is generally higher trained in epidemiology and infectious diseases than local health department staff and they are routinely involved in quality control of incoming case reports and also training and supervision of local health departments' staff.

Classification of the clinical picture resulted in significantly better results in the univariate analysis if the relevant case definition had a check-list format of the clinical picture (for diseases notifiable only in eastern states) as opposed to case examples of diseases for which the relevant case definition had a narrative description of the clinical picture (as in the old version of the national case definitions which participants had used as a reference). The dominant effect of the disease-variable, however, made this association disappear in the multivariate model. The most convincing explanation for this effect is, that after initial experience with the national case definitions, RKI had already changed the way of defining the clinical picture when creating case definitions for the diseases only notifiable in Eastern German states: The clinical picture was now defined in a clear check-list of signs and symptoms, instead of an unspecific mentioning of various possible signs and symptoms, such as in the first edition of the IfSG case definitions. Based on the findings of this study we have in the meanwhile applied this principle of a clear check-list in the second edition of the IfSG case definitions.

The results of the study were integrated in a case example book, which contains each of the 68 case examples followed by the required gold standard classification, descriptive statistics of the responses and a commentary interpreting these results and explaining the required gold standard. This case example book was mailed to all participants after termination of the study and is also available on the RKI website [22].

## Conclusion

All the observed quantitative effects and their propagated explanations merge into the one main conclusion: Case definitions must be very carefully formulated in order to assure their unambiguous interpretation by local health department personnel. The detailed evaluation of our study has resulted in a substantially revised edition of the German case definitions [23,24]:

• We rephrased the case definitions in a check-list format indicating clearly how many of the symptoms and signs had to be fulfilled in which combination.

• Some diseases previously jointly described in one case definition were defined separately (Dengue was separated from other haemorrhagic fever; hemolytic uraemic syndrome was created new, separately from EHEC and *Shigella*.)

• We rephrased the definitions in a way that for serologic confirmation the necessity for two samples is clearly apparent at the beginning of the phrase.

• The material in which the pathogen has to be detected is now highlighted and is only listed if it is relevant for the case definition.

• A glossary now defines the expressions that are being used repeatedly in the case definitions

• The case definitions are now limited to criteria relevant for the decision process. All additional explanatory information is clearly indicated as such in a separate section of the case definition

• The evidence type "epidemiological confirmation" was completely redesigned and replaces the previously used term "epidemiological link". The accepted types of epidemiological links are now specified individually for each case definition.

One practical implication, that is supported by this analysis is, that software used at the local health department must be designed with strict accordance to the case definitions using identical terminology and structuring which would have been more easily archived if all local health departments had been equipped with one identical software system developed within or under supervision of one institution. Possibly other countries in the process of developing or implementing new electronic surveillance systems might want to learn form this experience [21,25].

The case example book, which resulted from this study, constitutes a detailed feed back for the participants of the study and is now being used as training material for public health nurses.

We have demonstrated that rigorous reduction of case definitions to testable yes/no-criteria in a check-list format is likely to improve their reliability. Reducing the differential diagnostic complexity of a disease to a limited number of yes/no-criteria, is a major challenge, but it also carries the benefit of facilitating computerized testing algorithms for quality control and for case classifications.

As the reliability of epidemiologic surveillance largely depends on the reliability of its case definitions, it is essential to create and revise case definitions based on systematic evaluations [9]. Most of the basic principles for the revision of the German case definition edition deducted from this analysis may also be applicable for case definitions in other countries (such as the United States, Ireland, Sweden, Mexico) or international networks (EU, WHO) as they share the same structural and editorial characteristics that we identified to be problematic in the first edition of the German case definitions [4,7,8,26,27]. We therefore believe that our findings are highly relevant for many national and international surveillance systems.

## Competing interests

The author(s) declare that they have no competing interests.

## Authors' contributions

GK conceived of the study and is responsible for the design of the study, the creation of case examples and the gold standard definition. He supervised the study and is responsible for the analysis and interpretation of the data and the literature research.

BB is responsible for the pilot testing of case examples and survey, the study management, data entry and data management, and participated in the data analysis.

DA participated in the statistical analysis.

HC participated in the management of the data, the design of the study and the data analysis.

JB participated in the definition of the gold standard, the analysis and the interpretation of data and is responsible for the revision of new case definitions.

All authors read and approved the final manuscript.

## Pre-publication history

The pre-publication history for this paper can be accessed here:



## Figures and Tables

**Table 1:** Examples of the RKI-case definition for Salmonellosis in the original 2001 Version and the revised 2004 Version

**Version 2001 (reference 14)**
Clinical picture:
Clinical picture compatible with salmonellosis, characterized by diarrhea, abdominal pain, malaise, vomiting, fever. *Salmonella *can also cause infections outside the intestinal tract (for example: arthritis, endocarditis, pyelonephritis, septicaemia).
Laboratory diagnosis:
Isolation (culture) of pathogen from stool or other clinical material (e.g. blood, urine). The identification of serogroup has to be attempted.

**Version 2004 (reference 13)**

Clinical picture
• Clinical picture of acute salmonellosis, defined as at least one of the following four symptoms: diarrhea*
• cramp-like abdominal pain
• vomiting
• fever*
additional information:
*Samonella *can also cause generalized (septicemia) and localized infections outside the intestinal tract (for example: arthritis, endocarditis, pyelonephritis). These should in case of an acute infection also be reported. The reactive arthritis, which may also be caused by *Salmonella *infection, is not to be reported.
Laboratory diagnosed
Positive finding using the following method:
• Direct detection of pathogen: isolation of pathogen (culture)
Additional information: Results of identified serogroup and lysotype should also be reported.
Epidemiological confirmation
Epidemiological confirmation, defined as at least one of the following three constellations while taking into account the incubation period (about 6 to 72 hours):
• Epidemiological link to another laboratory-diagnosed human infection through
○ Person-to-person transmission OR
○ Same source of exposure (e.g. animal contact*, food*)
• Consumption of food (including drinking water), for which *Salmonella *spp. was laboratory-detected in non-consumed food.
• Contact to animal (e.g. poultry) with a laboratory-detected infection, or contact to its secretions or consumption of its products (e.g. eggs).
* terms marked with an asterix are defined in more detail in a glossary of the case definitions

**Table 2:** Distribution of case examples in different groups of participants

**Group 1**	**Group 2**	**Group 3**	**Group 4**
**Local health departments in West German States**			
**Set A**	**Set B**	**Set C**	**Set D**
Salmonellosis *	Salmonellosis *	Salmonellosis *	Salmonellosis *
Hepatitis B	Measles^+^	Creutzfeldt-Jakob-Disease (CJD)^+^	Meningococcal meningitis
Hepatitis C	Adenovirus conjunctivitis	Pathogenic *E. coli *enteritis	Influenza
Tuberculosis^+^	Polio^+^	EHEC** (enterohaemorrhagic *E. coli*)	Haemorrhagic fever

**Local health departments in East German States**			
**Set E**	**Set F**	**Set G**	**Set H**

Like Set A, plus:	Like Set B, plus:	Like Set C, plus:	Like Set D, plus:
Pneumococcal disease*	Borreliosis*	Rubella*	Viral meningitis*

Four different case examples were presented for each disease:

* The *Salmonella *case examples consisted of two case examples of *Salmonella enterica *spp., 1 case example of *Salmonella *Paratyphi and one case example of a salmonellosis-like enteric disease without identified pathogen.

** The EHEC case examples consisted in three case examples of EHEC and one case example of enterohaemorrhagic shigellosis.

^+ ^Diseases for which classification A-E are reportable to the next public health level; for all other diseases only classifications B-E are reportable to the next level.

**Table 3:** Disease specific reporting precision according to disease of the case examples. Reference variable: *Salmonellosis *case examples; outcome variable: reporting precision (n= 5995)

**Disease**	Odds Ratio *	**Confidence Interval**
Salmonellosis	-	-
Tuberculosis	0.678	0.477 – 0.965
Measles	0.375	0.277 – 0.509
Hepatitis C	0.277	0.208 – 0.368
Pathogenic *E. coli *enteritis	0.229	0.173 – 0.304
Adenovirus conjunctivitis	0.173	0.130 – 0.230
Haemorrhagic fever	0.161	0.123 – 0.210
Meningococcal meningitis	0.153	0.117 – 0.201
EHEC (enterohaemorrhagic *E. coli*)	0.099	0.076 – 0.129
Influenza	0.093	0.071 – 0.121
Hepatitis B	0.057	0.043 – 0.075
Creutzfeldt-Jakob-Disease (CJD)	0.012	0.008 – 0.017
Polio	0.008	0.005 – 0.013

+ The higher the odds ratio the higher the agreement with the gold standard. E.g. the chance of agreement with the gold standard was only two third (OR = 0.678) for tuberculosis case examples compared to Salmonella case examples.

* The four salmonella-like examples consisted in 2 examples of *Salmonella enterica*, one example of *Salmonella *Paratyphi and one example of an enteric disease with unidentified pathogen.

** The four EHEC-like case examples consisted in four causes by EHEC and one cause by *Shigella*.
